# 

**Published:** 1898-09

**Authors:** 


### CONTENTS.

| | PAGE |
|---|---|
| **Melancholia.** By Lionel A. Weatherly, M.D., C.M. Aberd., M.R.C.S. Eng. | 197 |
| **Hysterical Paroxysmal Œdema.** By F. H. Edgeworth, M.B., B.C. Cantab., B.Sc. Lond. | 206 |
| **Fifty Consecutive Intra-Abdominal Operations.** By James Swain, M.S., M.D. Lond., F.R.C.S. Eng. | 211 |
| **A Case of Laparotomy in which a Large Pyosalpinx simulating a Suppurating Tubo-ovarian Cyst was removed.** By J. Lacy Firth, M.S. Lond., F.R.C.S. Eng. | 229 |
| **Progress of the Medical Sciences:** | |
| **Medicine.** R. Shingleton Smith, M.D. | 234 |
| **Surgery.** W. H. Harsant | 238 |
| **Dermatology.** Henry Waldo, M.D. | 241 |
| **Reviews of Books** | 246 |
| **Notes on Preparations for the Sick** | 276 |
| **The Library of the Bristol Medico-Chirurgical Society** | 279 |
| **Local Medical Notes** | 282 |
| **Scraps** | 283 |

